# Structure of a Highly Active Cephalopod S-crystallin Mutant: New Molecular Evidence for Evolution from an Active Enzyme into Lens-Refractive Protein

**DOI:** 10.1038/srep31176

**Published:** 2016-08-08

**Authors:** Wei-Hung Tan, Shu-Chun Cheng, Yu-Tung Liu, Cheng-Guo Wu, Min-Han Lin, Chiao-Che Chen, Chao-Hsiung Lin, Chi-Yuan Chou

**Affiliations:** 1 Department of Life Sciences and Institute of Genome Sciences, National Yang-Ming University, Taipei 112, Taiwan

## Abstract

Crystallins are found widely in animal lenses and have important functions due to their refractive properties. In the coleoid cephalopods, a lens with a graded refractive index provides good vision and is required for survival. Cephalopod S-crystallin is thought to have evolved from glutathione S-transferase (GST) with various homologs differentially expressed in the lens. However, there is no direct structural information that helps to delineate the mechanisms by which S-crystallin could have evolved. Here we report the structural and biochemical characterization of novel S-crystallin-glutathione complex. The 2.35-Å crystal structure of a S-crystallin mutant from *Octopus vulgaris* reveals an active-site architecture that is different from that of GST. S-crystallin has a preference for glutathione binding, although almost lost its GST enzymatic activity. We’ve also identified four historical mutations that are able to produce a “GST-like” S-crystallin that has regained activity. This protein recapitulates the evolution of S-crystallin from GST. Protein stability studies suggest that S-crystallin is stabilized by glutathione binding to prevent its aggregation; this contrasts with GST-σ, which do not possess this protection. We suggest that a tradeoff between enzyme activity and the stability of the lens protein might have been one of the major driving force behind lens evolution.

Crystallins are soluble proteins in eye lenses that play an important role in the maintenance of lens transparency and optical clarity^1^. Their aggregation can result in the formation of cataracts, which are the most common cause of blindness worldwide^2^. Up to the present, the only treatment for this is the surgical removal of cataractous lens, albeit it has been reported very recently that lanosterol may reverse aggregation of crystallin^3^. Interestingly, some taxon-specific crystallins have been found to have structures that are related to cytosolic housekeeping enzymes^4^,^5^. How lens crystallins evolved from such housekeeping enzymes is a most important question and answers to this question will greatly help our understanding of protein function, folding, and stability^6^.

Among the crystallins, S-crystallin in the cephalopod lens has been shown to have an amino acid sequence similar to that of glutathione S-transferase (GST)^6^,^7^,^8^, which catalyzes the conjugation of glutathione (GSH) to various endogenous and xenobiotic electrophilic compounds and is a major detoxification mechanism of biological systems^9^,^10^,^11^,^12^ (Extended data Fig. 1). Previous studies have led to a model that involves multiple gene duplications and exon shuffling to allow the evolution from GST to S-crystallin^4^,^7^,^8^ and this process then creates a diverse and large family of S-crystallins. There are about ten, twenty four and four different S-crystallins in the lens of the squids, *Nototodarus sloanii* and *Loligo opalescens*, and *Octopus vulgaris* (the West Pacific octopus), respectively^7^,^13^,^14^. The cDNAs encoding these S-crystallins have nucleotide sequence identities ranging from 58% to 98%, while their proteins have identities ranging from 46% to 99% at the amino acid sequence level^7^. Among these proteins, “short-loop” S-crystallins (SL11, S4 and Cry9) are expressed less in the lens and are more GST-like, while “long-loop” S-crystallins are dominantly expressed in the lens and are enzymatically inactive^7^,^15^. Up to the present there has been no direct structural information available that allows us to understand the reason for the differences.

## Results and Discussion

**Figure 1:**
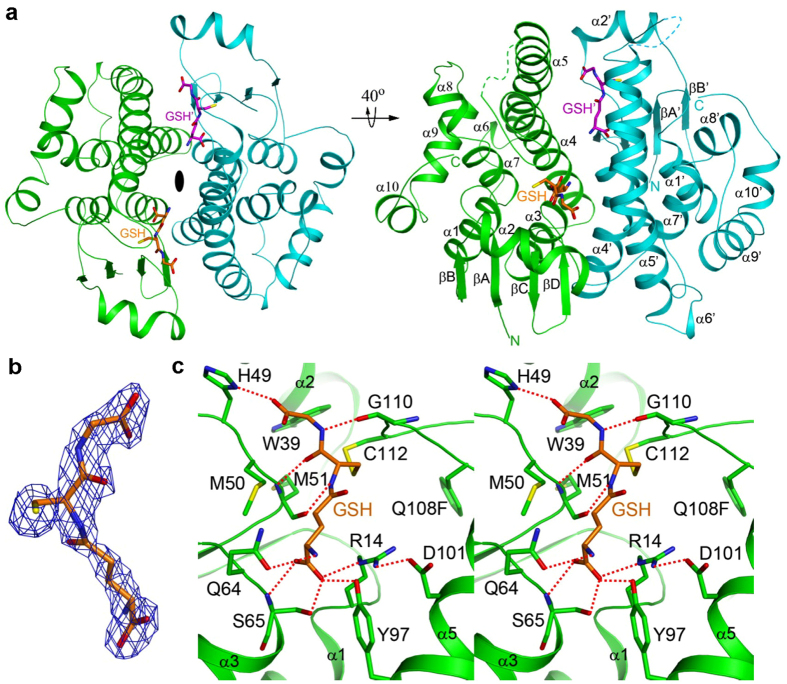
Crystal structure of the octopus S-crystalin Q108F mutant in complex with GSH. (**a**) A schematic drawing of the structure of the S-crystallin dimer (in green and cyan) in complex with one of two substrates, GSH (in orange and magenta), in two views. Residues (117 to 124), which are missing in the α4-α5 loop, are indicated by dashed lines. The 2-fold axis of the dimer is indicated by the black oval. (**b**) The *F*_*o*_*-F*_*c*_ electron density at 2.35 Å resolution of GSH contoured at 3.5σ. (**c**) A stereo pair showing the detailed interactions between the GSH and the active site of S-crystallin. Ion-pair and hydrogen-bonding interactions are indicated by red dashed lines. All structural figures were produced using PyMOL (http:/www.pymol.org).

In the present study we overexpressed several long-loop S-crystallins of *Octopus vulgaris* in *Escherichia coli*. Only one of them, named OctS4, could be expressed and purified to homogeneity. However, efforts to crystallize the wild-type OctS4 were unsuccessful. Therefore, we modified the wild-type sequence by creating a number of mutants that are believed to be related to the binding of GSH or of electrophilic compounds (Extended data Fig. 1). After a significant number of trials, a highly active mutant, Q108F, was successfully expressed and crystallized in the presence of GSH. We determined the crystal structure of the S-crystallin Q108F mutant at 2.35 Å resolution (Fig. 1 and Extended data Table 1). A monomer of S-crystallin forms an asymmetric unit within the crystal, and a dimer of the protein that is similar to GST can be generated using a crystallographic 2-fold symmetry axis (Fig. 1a and Extended data Fig. 2a). Approximately 1600 Å^2^ of the surface area of each monomer is buried in the interface of this dimer, comparing to 1300 Å^2^ for the squid GST-σ^16^ with 38% sequence identity to S-crystallin. Besides, the dimerization of S-crystallin and several of its mutants was confirmed by analytical ultracentrifugation (Extended data Fig. 2).

**Figure 2:**
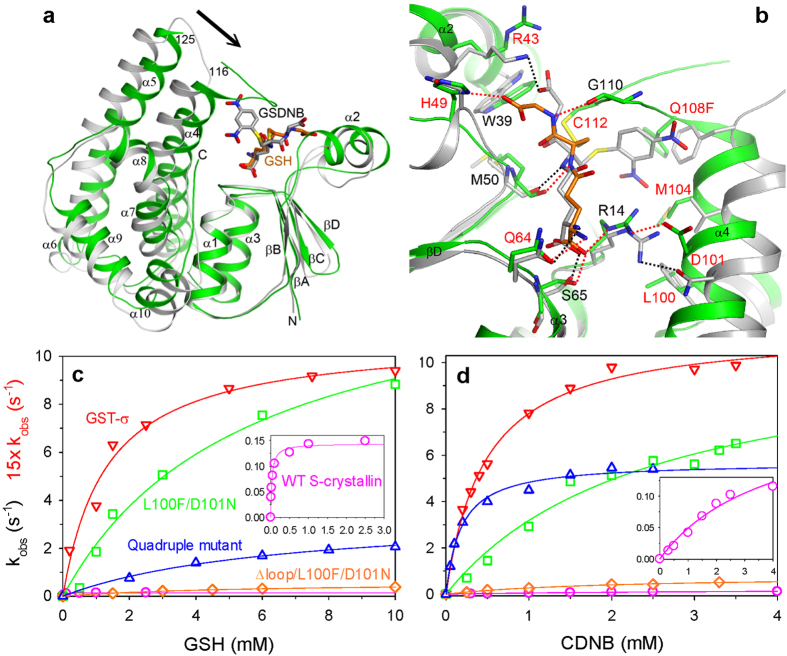
Structural and biochemical characterizations of S-crystallin. (**a**) An overlay of the structure of the S-crystallin Q108F mutant in complex with GSH (green) against GST-σ in complex with GSDNB (grey)^16^. The arrow indicates the conformational differences in the α4 and α5 helices. (**b**) Overlay of the active sites of the two structures. Residues labeled in red were selected for mutagenesis. (**c,d**) The GST activity of S-crystallin. Plot of the rate constant as a function of the concentration of two substrates GSH (**c**) and CDNB (**d**) for the wild-type (magenta) and various S-crystallin mutants, the L100F/D101N double mutant (green), the ∆loop/L100F/D101N double mutant (orange), and L100F/D101N/M104V/Q108F quadruple mutant (blue). The activity of GST-σ from octopus liver tissues (red) was used as a positive control. Insets show plots of the wild-type S-crystallin at a suitable scale.

The reported crystal structures of GST-σ are in complex with either 1-(S-glutathionyl)-2,4-dinitrobenzene (GSDNB) or S-(3-iodobenzyl) glutathione (GSBzI)^16^. In contrast to the above, we observed the presence of a GSH molecule within the active site of S-crystallin, located between the N-domain (βA to α3) and C-domain (α4 to α10) of the protein (Fig. 1b). There is a disulfide-bond between the thiol group of the GSH and residue Cys112 of S-crystallin. In addition, there is a polar interaction network (Fig. 1c) that consists of the glutamyl group of GSH interacting with residues Arg14, Gln64, Ser65 and Tyr97, the cysteinyl group of GSH interacting with residue Met51 and the glycinyl group of GSH interacting with residues His49 and Gly110. The two domains of S-crystallin are closer compared to those of GST-σ in complex with GSDNB; although the r.m.s distance between equivalent Cα atoms of the two structures is 1.3 Å (Fig. 2a). When their N-domains are superposed, a difference of 3.5° can be seen in the orientation of their C-domains.

Compared with the binding of GSH to S-crystallin, GSDNB has fewer hydrophilic interactions with GST-σ (Fig. 2b). GSDNB has no interaction with residue Arg13 of GST-σ, which is equivalent to residue Arg14 of S-crystallin. In addition, due to the lack of a long loop between the α4 and α5 helices (residues 112 to 122), the glycinyl group of GSDNB does not interact with the C-domain of GST-σ, while its dinitrobenzyl group does have hydrophobic contact with Phe106. Similarly, the structure of GST-σ in complex with GSBzI indicates that the iodobenzyl ring of GSBzI is located in a hydrophobic pocket that consists of residues Phe98, Val102 and Phe106 (Extended data Fig. 3). Like other GSTs^17^, the different binding modes of the GSDNB and the GSBzI to GST-σ imply a more flexible active site that favors catalysis. In contrast, the equivalent residues in S-crystallin are Leu100, Met104 and Gln108, respectively (Fig. 2b). These differences result in a collapsed pocket that may disfavor the binding of aromatic compounds. We characterized the GST activity levels of S-crystallin and its various mutants in order to evaluate the role of the above residues. Octopus GST-σ was purified from the digestive gland and then used as a standard for enzyme activity (Fig. 2c,d). The GST catalytic activity (k_cat_) of the wild-type S-crystallin is 0.24 s^−1^, which is about the same to that of the S-crystallins purified from octopus lens^18^ but only ~1/700, of that of GST-σ (it will be ~1/6000 if compared their catalytic efficiency by k_cat_/K_m,CDNB_) (Table 1). Consistent with our structural observations, we found that the apparent binding affinity of 1-chloro-2,4-dinitrobenzene (CDNB) to S-crystallin is lower than that with GST-σ, with an 8.3-fold increase in the K_m,CDNB_. By way of contrast, the apparent binding affinity of GSH with S-crystallin is significantly tighter than that with GST-σ, with a 43-fold decrease in K_m,GSH_.

Kinetic analysis indicated that three single mutants, L100F, M104V and Q108F, have a higher k_cat_ than that of wild-type S-crystallin with 10- to 44-fold increases, respectively (Extended data Fig. 4c,d and Extended data Table 3). The K_m,GSH_ of the L100F and M104V mutants of S-crystallin have 37-fold and 83-fold increases and are similar to that of GST-σ, while K_m,CDNB_ of the M104V mutant shows a significant decrease and is almost identical to that of GST-σ (0.65 mM versus 0.47 mM). Interestingly, although the Q108F mutant of S-crystallin possesses the highest k_cat_ among the three mutants, its K_m,GSH_ is also very high and we were not able to estimate a reliable K_m,CDNB_ due to unsaturated kinetic assays. This indicates that the mutation of Gln108 to Phe is beneficial for catalysis but is detrimental to the binding of the two substrates. These results suggest that, in order to obtain a more “GST-like” S-crystallin, no single mutant is suitable, but rather multiple mutations are necessary (see below).

Another difference between S-crystallin and GST-σ occurs with residue Asp101, which is at the active center (Asn99 in GST-σ) (Fig. 2b). Although both residues are located in the vicinity of an arginine residue, the “charge-charge” interaction between Asp101 and Arg14 in S-crystallin is likely to diminish the important function of arginine residue, which is to stabilize the negatively charged intermediate state (the Meisenheimer complex) during catalysis^19^. Indeed, the mutation of Asp101 to Ala or Asn resulted in a minor increase in k_cat_ in parallel with a decrease in the K_m,CDNB_ of S-crystallin; albeit a 13-fold to 87-fold increase in K_m,GSH_ (Extended data Fig. 4c,d and Extended data Table 3). Mutation of other residues involved in the binding of GSH, such as Arg43 (to Lys) and His49 (to Asn) (Fig. 2b), resulted in no significant differences in GST activity (Extended data Fig. 4a,b and Extended data Table 3). On the other hand, based on our structural observations and our kinetic assays, the highly conserved residue Gln64 would seem to be essential for the binding of GSH and for catalysis (Fig. 2b and Extended data Table 3).

Next we focused on four key residues of S-crystallin, Leu100, Asp101, Met104 and Gln108, mutation of which resulted in large changes in catalysis or binding. The GST activity of the various double, triple and quadruple mutants of S-crystallin was characterized in order to delineate their mutual effects (Fig. 2c,d, Extended data Fig. 4e,f and Extended data Table 3). The results suggest that, compared with of those from the single mutants, all double and triple mutants of S-crystallin have comparable or even higher k_cat_ values, while there were no significant differences in the K_m_ with GSH or CDNB. Furthermore, the quadruple mutant of S-crystallin, L100F/D101N/M104V/Q108F, had the lowest K_m,CDNB_ (0.18 mM) and the highest K_m,GSH_. Although there was only a 23-fold increase in activity for some unknown reason, the catalytic efficiency (k_cat_/K_m,CDNB_) has a 518-fold increase (Table 1). Nevertheless, based on the switch in the two substrate-binding affinities (increased K_m,GSH_ and decreased K_m,CDNB_) and the significant recovery of GST activity and catalytic efficiency, the double mutant D101N/Q108F and the quadruple mutant of S-crystallin would seem to result in a GST-like S-crystallin that has recovered GST activity and probably a more flexible active site^17^. In addition, another double mutant of S-crystallin, D101A/Q108F, showed the highest enzymatic activity in these mutants, which is only a 7-fold lower than that of GST-σ (Extended data Table 3).

Our structure demonstrates that the long loop between the α4 and α5 helices of S-crystallin is able to bind to GSH through disulfide-bonding interaction via residue Cys112 and via a hydrogen bonding interaction with residue Gly110 (Fig. 1c). Next we characterized the GST activities of the C112G and loop-deletion mutants of S-crystallin in order to evaluate the effect of these changes. Interestingly, the mutation of Cys112 to Gly did not influence the binding affinity of GSH to S-crystallin; although the GST activity of the mutant is 3-fold lower than that of the wild-type (Extended data Fig. 4a,b and Extended data Table 3). This indicates that the disulfide bonding between S-crystallin and GSH is not essential for binding. This also helps to explain the observation that the equivalent residue in most other S-crystallins is a glycine^7^,^14^ (Extended data Fig. 1).

Not surprisingly, the deletion of the long loop (residues 112–122) resulted in an obvious decrease in the apparent binding affinity of GSH as with a 73-fold increase in K_m,GSH_ but a similar k_cat_, compared to the wild-type S-crystallin (Extended data Fig. 4g,h and Extended data Table 3). These features prompted us to make a mutant that consisted of the four key residues changed previously together with the loop deletion in order to generate a putative “ancestral” protein. However, other than the Δloop/Q108F mutant of S-crystallin, the other mutations together with the loop deletion resulted in a low level of recovery of activity and poor binding affinity with GSH (Fig. 2c,d, Extended data Fig. 4g,h and Extended data Table 3). Unlike the Q108F mutant alone form of the protein, the K_m,CDNB_ of the Δloop/Q108F mutant of S-crystallin can be precisely determined to be 1.9 mM. This suggests that existence of the long loop not only enhances the binding of GSH to S-crystallin but may interfere with the binding of electrophilic compounds.

**Figure 3:**
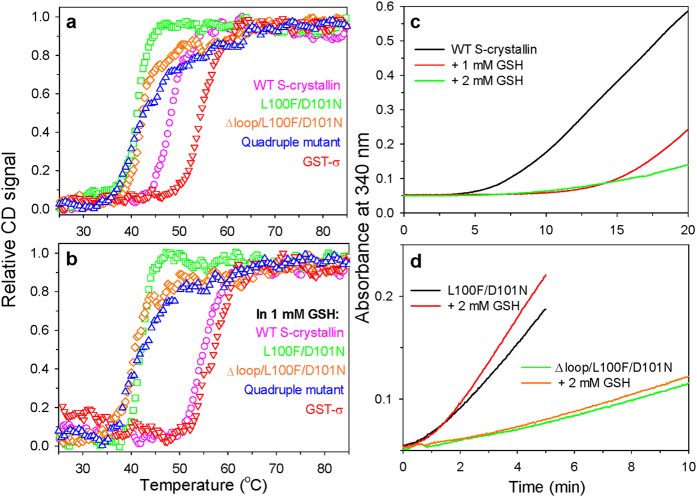
Protein stability of S-crystallin and its mutants by heat denaturation (**a,b**) and chemical denaturation (**c,d**). Plots of the relative CD signal from the ellipticity at 222 nm as a function of the temperature for the wild-type (magenta) and various S-crystallin mutants, the L100F/D101N double mutant (green), the ∆loop/L100F/D101N double mutant (orange), and the L100F/D101N/M104V/Q108F quadruple mutant (blue) without (**a**) or with (**b**) 1 mM GSH. The thermal stability of GST-σ (red) without or with 1 mM GSH was also measured. The protein concentration was 7.2 μΜ. The results were fitted in order to calculate their T_m_ of each mutant. which are shown in Table 2. (**c,d**) Denaturant-induced light scattering of S-crystallin (**c**) and its mutants (**d**). The aggregation traces of S-crystallin in 3.5 M urea without or with GSH at 1–2 mM were observed by light-scattering at 340 nm. The protein concentration was 10 μM. The assays were repeated twice to ensure reproducibility.

To understand the mechanisms by which changes in gene sequence generate shifts in function and therefore phenotype^20^,^21^, it is necessary to analyze how the changes in protein structure mediate the effects that the mutations have on function^22^. Interestingly, a long-loop S-crystallin (OctS4) shows a significant increase in apparent GSH binding affinity compared to that of GST-σ. To evaluate the possible biological significances of such strong GSH binding, the thermal stability of S-crystallin without or with GSH was determined using circular dichroism spectrometry (Fig. 3a,b). In the presence of GSH, the melting temperature (T_m_) of S-crystallin was higher by 7 °C than that of the protein in the absence of GSH (Table 2). Again, the mutation of Cys112 to Gly did not influence the T_m_ enhancement in the presence of GSH (Extended Data Fig. 5a,d and Extended Data Table 4). This indicates that the disulfide bonding between S-crystallin and GSH is not essential for this protection. Furthermore, denaturant-induced aggregation of S-crystallin was also observed using light-scattering analysis (Fig. 3c). Our results suggested that S-crystallin aggregation is prevented by the presence of GSH in a dose-dependent manner. By way of contrast, GST-σ showed only a 1.7 °C increase in T_m_ in the presence of GSH; although the T_m_ of the free GST-σ is higher than that of the free S-crystallin. It indicated that GSH may not benefit to stabilize the GST-σ. Differently, previous studies have suggested that GSH binding is required to stabilize GSTP1-1 and *Plasmodium falciparum* GST^23^,^24^,^25^.

The structural protection and aggregation prevention afforded to S-crystallin by GSH suggested that in the lens S-crystallin may bind to GSH and that this contributes to the overall stability of lens proteins. In addition, this explains why S-crystallin from the lens was unable to bind to a GSH affinity column in earlier studies^19^,^26^,^27^. Interestingly, all animals have an abundance of reducing agents in the lens, including GSH (2–10 mM), in order to maintain the redox potential of the lens^28^. Traditionally, the high GSH content in lens is believed to protect free thiols, which allows the structural integrity and/or proper biological functioning of various enzymes to be maintained^28^. Aging or damaged lenses show a diminished size of GSH pool and accelerating cataract. Previous studies have suggested that glutathiolation of human gamma crystallin is able to enhance its degradation by a proteasome system^29^. Here our novel finding is that GSH can also be used as a ligand of S-crystallin in order to prevent its aggregation and possible cataract formation in the cephalopod lens.

If we considered the situation of S-crystallin in the lens, it is important for this protein to capture GSH for as long as possible and to minimize its catalytic activity, otherwise the GSH will be released as a product conjugate. This is likely to be a driving force that provided the impetus for the ancestral protein to evolve in a manner that weaken its binding affinity for electrophilic compounds by mutating residues 100, 104 and 108 and that interferes with the formation of the intermediate complex by mutating residue 101. In addition, inserting the long loop between α4 and α5 helices is also able to enhance the binding of GSH while at the same time interfering with the binding of electrophilic compounds. We believe that these mutations may play crucial roles in the evolutionary trajectory of S-crystallin. Similarly, it has been previously reported that the old human GST, GSTT2-2, shows a low affinity for GSH, compared to the more recently evolved human Alpha, Mu, and Pi GSTs^17^. It is possible to speculate that GST and S-crystallin may be evolved from a common GST ancestor under the same evolutionary pressure, i.e. in the direction of a decrease in K_m_ for GSH.

The thermal stabilities of the various mutations with or without the long α4-α5 loop were also characterized. Other than the Q108F mutant of S-crystallin, all the mutants that showed an increased K_m_ or a recovery in activity were found to have a lower T_m_ without GSH (Fig. 3a,b, Extended data Fig. 5 and Extended data Table 4). This suggests that the original residues and the insertion of the long loop of S-crystallin improve the stability of the protein. Furthermore, other than the L100F and D101N mutants of S-crystallin, all the other mutations were found to have lost the protective effect of GSH as there were no T_m_ increases when GSH was present. Based on these findings, we concluded that the existence of the long loop and the collapse of the binding pocket are both required for S-crystallin to be able to be protected in terms of stability when GSH is present. Furthermore, the mutation of the residue 101 from Asn to Asp is able to increase the stability of S-crystallin and this occurs because of a strengthening of the GSH protection effect.

To recapitulate the evolutionary trajectory of S-crystallin from its putative ancestral protein, GST or a GST like protein^8^, it was possible to create various different proteins by mutation of “GST” towards a more “S-crystallin”-like protein and by mutation of “S-crystallin” towards a “GST”-like protein. We have successfully generated a GST-like S-crystallin that has a 100-fold increase in activity and shows a switch in substrate-binding affinity. As the next step, using the full gene synthesis, we created a “S-crystallin”-like GST, by a quadruple mutation, F98L/N99D/V102M/F106Q, and the insertion of the long loop into the α4-α5 helices of the GST-σ sequence (Extended data Fig. 6). However, although the S-crystallin-like GST protein showed a 120-fold reduction in catalytic activity and an unsaturated pattern for the binding of CDNB (Table 1); this protein failed to gain the ability to be protected by GSH (Table 2). Mutations of other residues involved in the binding of GSH may be necessary to retain GSH in the protein.

Another concern when deciphering the evolution of S-crystallin is whether we can find a orthologous S-crystallin that is close to ancestral protein, in other words, a “molecular fossil”^8^. There are many different kind of S-crystallins found in the cephalopod lens as a result of gene duplication and/or the recruitment of novel genes into this morphogenetic pathways^7^,^14^,^15^. Previous studies have suggested dividing S-crystallins into two groups, the short-loop group, which is less expressed and the long-loop group, which are the dominant forms of S-crystallin. Based on sequence alignment, the short-loop crystallins are considered to be the earliest descendants from the ancestral gene^7^. In the present studies, we have identified one long-loop S-crystallin, SL-18, that is different to the others because it preserves residues 100 and 101 as Phe and Asn, respectively (Extended data Fig. 1). Actually, the short-loop S-crystallins (SL11, LopS4 and Cry9) from three species of squids also preserve the two residues^7^,^13^,^15^. We chose two mutants, Δloop/L100F/D101N and L100F/D101N, and used these to mimic the short-loop and SL18 S-crystallins, respectively (Figs 2c,d and 3). The long-loop deletion resulted in the loss of GSH protection and the ability to prevent aggregation (Table 2 and Fig. 3d); although the GST activity of short-loop mimic showed a 3.3-fold increase (Table 1). This slight increase in activity is compatible with the findings of previous studies wherein lysate containing expressed short-loop-crystallin showed minor GST activity^7^. On the other hand, the SL18 mimic protein showed significantly high GST activity (a 59-fold increase) as well as lower thermal stability and the inability to prevent aggregate (Tables 1 and 2 and Fig. 3). For the first time we have provided experimental evidence showing that a modern S-crystallin with high GST activity do exist and that this may be advantageous to help protect other proteins in cephalopod lens when GSH is present.

**Figure 4:**
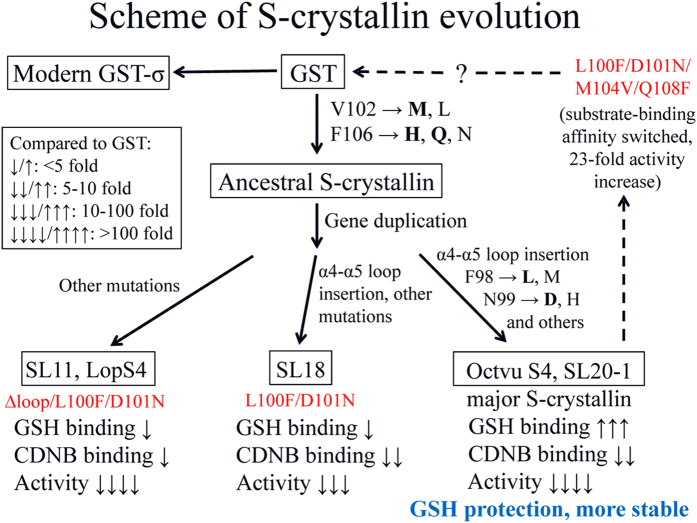
Putative evolution of cephalopod S-crystallin. The three different groups of S-crystallin are separated by the mutations at two residues F98 and N99 and by the insertion of a long loop between the α4 and α5 helices. Two other residues, V102 and F106, can be used to separate GST and the ancestral S-crystallin. With a longer α4-α5 loop and the four “key” mutations, major S-crystallins lose their GST activity but gain significantly increased GSH binding affinity. The binding of GSH to S-crystallin enhances its protein stability, which is beneficial for the life-span of the lens and the survival of cephalopods. According to previous studies^29^, there is between 2 mM and 10 mM GSH in animal lenses.

Here we have provided a schematic summary that demonstrates the structural/functional variations that affect duplicated S-crystallins (Fig. 4). To have a major dominant modern S-crystallins that has GSH protection, there are three groups of mutations needed: those affecting the binding pocket of the electrophilic compound (GST’s Phe98, Val102 and Phe106 residues), those affecting intermediate state stabilization (Asn99), and the presence of a long-loop insertion between the α4 and α5 helices. Sequence alignment suggests that all modern S-crystallin have mutations at Val102 and Phe106, which indicates that these events have happened quite early in the ancestral S-crystallin before gene duplication occurred. Later, Phe98 and Asn99 were mutated resulting in a further loss of enzyme activity with only the short-loop (SL11, LopS4, Cry9) and one long-loop S-crystallin (SL18) still retaining these two residues. In a final step, there is an insertion of the various long-loop, which produces the goal of GSH protection while making the protein more stable.

Overall, our findings have revealed a novel structure for the cephalopod S-crystallin and provided crucial evidence that greatly aids our understanding of the evolution of S-crystallin. Loss of GST enzyme activity is linked to gaining the enhanced protein stability of S-crystallin and this occurs via GSH binding. These changes can be considered to be the major driving force during the evolution of S-crystallin. Moreover, the secret of the stability of crystallin may help us find a strategy that will allow us either to prevent or treat cataracts.

## Methods

### Protein expression and purification

The west Pacific octopus (*Octopus vulgaris*) was purchased from local fish market and keep on ice during the transfer. The cDNA of the octopus was prepared by RNA extraction from lenses according to standard procedures^14^. One of the major S-crystallin cDNAs was then subcloned into pET-28a vector (Novagen). A fused protein with a N-terminal 6x His tag was then expressed by *E. coli* BL21 Rosetta (DE3) (EMD Millipore). Cultures were incubated overnight at 20 °C and induced with 0.4 mM isopropyl-β-_D_-thiogalactopyranoside. The cell pellets were resuspended in lysis buffer (20 mM Tris, pH 8.5, 250 mM NaCl, 5% glycerol, 0.2% Triton X-100 and 2 mM β-mercaptoethanol), lysed by sonication and then centrifuged to remove any insoluble lysate. The remaining soluble proteins were then passed through nickel affinity beads (Qiagen) and then the bound expressed protein was released. This protein was subjected to further purified by gel-filtration (Sephacryl S-100; GE Healthcare) chromatography using a running buffer of 20 mM Tris, pH 8.5, 100 mM NaCl and 2 mM dithiothreitol. The purified protein was concentrated to 20 mg/ml, and the solution was supplemented with 5% (v/v) glycerol before being flash-frozen in liquid nitrogen and stored at −80 °C.

Octopus digestive gland GST was purified by a single GSH-Sepharose 4B affinity chromatography process that has been described previously^18^,^26^. Briefly, octopus digestive gland (20 g) from an octopus was homogenized with 10 mM phosphate buffer (pH 7.0) containing 2 mM mercaptoethanol and then subjected to ultracentrifugation at 4 °C and 80,000 g for 1 h. After filtration, the supernatant was further purified by GSH-Sepharose 4B affinity column. The fractions containing GST were pooled and supplemented with 5% (v/v) glycerol before being flash-frozen in liquid nitrogen and stored at −80 °C. All animal experiments conformed to the regulations drafted by Animal Protection Act in Taiwan and were approved by the laboratory animal center at National Yang-Ming University.

### Protein crystallization

Crystals of the S-crystallin Q108F mutant in complex with GSH were obtained at 22 °C by the sitting drop, vapor diffusion method. Initial crystal screens were set up after incubating the protein at 6 mg/ml in 7 mM GSH and 0.35 mM CDNB for 1 h. Poorly diffracting crystals were grown in 0.5% PEG8000, 0.1 M HEPES, pH 6.8 and 1.65 M ammonium sulfate in 3 days and these were used for microseeding. Single crystals of a hexagonal shape and with dimensions of 0.15 mm were obtained in less than a week. All crystals were cryoprotected in reservoir solution supplemented with 25% (v/v) glycerol and flash-cooled in liquid nitrogen.

### Data collection and structure determination

X-ray diffraction data were collected at 100 K on the SPXF beamline 13C1 at the National Synchrotron Radiation Research Center, Taiwan, R.O.C. using a ADSC Quantium-315r CCD detector (X-ray wavelength of 0.976 Å). The diffraction images were processed and scaled with the HKL-2000 package^30^. The crystals belong to space group P6_4_22, with cell dimensions of a = b = 114.3 Å, c = 63.9 Å, α = β = 90°, and c = 120°. There is one S-crystallin subunit in the crystallographic asymmetric unit.

The structure of S-crystallin was solved by the molecular replacement method by the program Molrep^31^ using a simulated model for octopus S-crystallin^19^ as the model. The atomic model was built using the program Coot^32^. Structure refinement was carried out using REFMAC^33^. The crystallographic information is summarized in Extended Data Table 1.

### Analytical ultracentrifugation analysis

The experiments were performed on an XL-A analytical ultracentrifuge using an An-60 Ti rotor (Beckman Coulter)^34^,^35^,^36^. The sedimentation-velocity experiments were performed using a double-sector epon charcoal-filled centerpiece at 20 °C with a rotor speed of 42,000 rpm. Protein solutions of S-crystallin and its mutants (0.025–1.0 mg/ml) and a reference buffer (50 mM phosphate, pH 6.5) were loaded into the centerpiece. The absorbance at 230 or 280 nm was monitored in continuous mode with a time interval of 300 s and a step size of 0.003 cm. Multiple scans at different time intervals were then fitted to a continuous c(M) distribution model using SEDFIT^37^,^38^.

### Mutagenesis and kinetic studies

Site-specific mutants were designed based on the available sequence alignment and structural information. The mutations were introduced using the QuikChange kit (Stratagene) and verified by DNA sequencing. The primers used for site-specific mutations are summarized in Extended Data Table 2.

The GST activity of S-crystallin was determined by measuring the conjugation of GSH and CDNB^26^,^39^. The formation of the glutathione conjugate was monitored continuously at 340 nm. A molar absorption coefficient of 9,600 M^−1^·cm^−1^ for the conjugate was used in calculations. The initial velocity study for pseudo-first order kinetic assay was performed by varying the concentration of GSH from 1 μM to 12 mM with a constant concentration of CDNB of 3.5 mM for the K_m,GSH_ calculation and by varying the concentration of CDNB from 0.1 to 3.8 mM with a constant concentration of GSH of 10 mM for K_m,CDNB_. The K_m_ was used as apparent binding affinity in the present studies. The non-enzymatic conjugation of GSH with CDNB (Extended data Fig. 4I,j) was corrected for during each assay.

### Thermal stability analysis

The unfolding of each protein was monitored by measuring the CD signal of 222 nm from 25 °C to 85 °C. The protein concentration was 0.2 mg/ml without or with 1 mM GSH. The data was analyzed using thermodynamic models by globally fitting the measurements to a two-state unfolding model in order to calculate the T_m_ of each protein^40^.

### Light-scattering measurement

Polymerization and aggregation of S-crystallin and its various mutants was measured by denaturant-induced light-scattering^41^. The protein was incubated with 3.5 M urea in the absence or presence of GSH at 30 °C. The protein concentration was 0.3 mg/ml. The light-scattering at 340 nm was continuously monitored by UV/VIS spectrometry (Jasco).

## Additional Information

**Accession codes**: Model coordinates and structure factors for the crystal structure of the octopus S-crystallin Q108F mutant was deposited in the Protein Data Bank (PDB code: 5B7C).

**How to cite this article**: Tan, W.-H. *et al.* Structure of a Highly Active Cephalopod S-crystallin Mutant: New Molecular Evidence for Evolution from an Active Enzyme into Lens-Refractive Protein. *Sci. Rep.* **6**, 31176; doi: 10.1038/srep31176 (2016).

## Supplementary Material

Supplementary Information

## Figures and Tables

**Table 1:** Steady-state kinetic parameters of S-crystallin, its various mutants and two GSTs.

Protein^a^	K_m,GSH_ (mM)^c^	K_m,CDNB_ (mM)^c^	k_cat_ (s^−1^)^c^
Wild-type S-crystallin	0.03 ± 0.004	3.9 ± 1.2	0.24 ± 0.05
L100F/D101N	5.5 ± 1.0	2.5 ± 0.7	14.1 ± 1.0
L100F/D101N/M104V/Q108F	6.5 ± 0.7	0.18 ± 0.02	5.6 ± 0.1
Δloop/L100F/D101N^b^	5.3 ± 0.4	2.0 ± 0.4	0.80 ± 0.07
Octopus GST-σ	1.3 ± 0.2	0.47 ± 0.04	173.6 ± 4.3
S-like GST	3.7 ± 0.4	unsaturated	1.45 ± 0.07

^a^The concentration of the six proteins was 1.1, 0.04, 0.1, 0.4, 0.001, and 0.2 μM, respectively. For the GSH titration, the concentration of CDNB was 3.5 mM. For the CDNB titration, the concentration of GSH was 10 mM.

^b^The loop between α4 and α5 helices (residue 112–122) was deleted and is designated as Δloop.

^c^Data were fitted to the Michaelis-Menten equation and the R_sqr_ values were 0.980 to 0.999, respectively. All the assays were repeated at least twice to ensure reproducibility. The K_m_ was used as apparent binding affinity in the present studies.

**Table 2:** Thermal stability of S-crystallin, its various mutants and two GSTs with or without 1 mM GSH.

Protein^a^	T_m_ (°C)	T_m_ in GSH (°C)	ΔT_m_1 (mutant-WT)	ΔT_m_2 (in GSH-apoform)
Wild-type S-crystallin	48.2 ± 1.0	55.2 ± 1.2	—	7.0
L100F/D101N	41.5 ± 1.2	41.9 ± 1.6	−6.7	0.4
L100F/D101N/M104V/Q108F	39.2 ± 1.3	39.9 ± 1.4	−9.0	0.7
Δloop/L100F/D101N	41.7 ± 2.0	40.3 ± 2.2	−6.5	−1.4
GST-σ	54.8 ± 0.9	56.5 ± 1.5	6.6	1.7
S-like GST	42.4 ± 1.9	41.7 ± 2.1	−5.8	−0.7

^a^The protein concentration was at 7.2 μM. The ellipticity at 222 nm was monitored while varying the temperature ranging from 25 °C to 85 °C. The results were fitted to the two-state unfolding model in order to calculate T_m_ of the various proteins.
