# Supplementary material for: Structure of a Highly Active Cephalopod S-crystallin Mutant: New Molecular Evidence for Evolution from an Active Enzyme into Lens-Refractive Protein
Source: Sci Rep. 2016 Aug 8;6:31176. doi: 10.1038/srep31176 (PMC4976375; doi:10.1038/srep31176)
Supplement: Supplementary Information [file srep31176-s1.pdf]

**Supplementary information for**

# Structure of a Highly Active Cephalopod S-crystallin Mutant: New Molecular Evidence for Evolution from an Active Enzyme into Lens-Refractive Protein

Wei-Hung Tan[1], Shu-Chun Cheng[1], Yu-Tung Liu[1], Cheng-Guo Wu[1], Min-Han Lin, Chiao-Che Chen, Chao-Hsiung Lin, and Chi-Yuan Chou*

Department of Life Sciences and Institute of Genome Sciences

National Yang-Ming University

Taipei 112, Taiwan

---

[1] These authors contributed equally to this work.

* Correspondence information for Chi-Yuan Chou

Phone: +886-2-28267168, FAX: +886-2-28202449

E-mail: cychou@ym.edu.tw

**References**


1 Gouet, P., Courcelle, E., Stuart, D. I. & Metoz, F. ESPript: analysis of multiple sequence alignments in PostScript. *Bioinformatics* **15**, 305-308, (1999).

2 Wu, C. G. *et al.* Mechanism for controlling the monomer-dimer conversion of SARS coronavirus main protease. *Acta Crystallogr D Biol Crystallogr* **69**, 747-755, (2013).

3 Lin, M. H. *et al.* Structural and functional characterization of MERS coronavirus papain-like protease. *J. Biomed. Sci.* **21**, 54, (2014).

4 Chou, C. Y. *et al.* Structural basis for catalysis and ubiquitin recognition by the severe acute respiratory syndrome coronavirus papain-like protease. *Acta Crystallogr D Biol Crystallogr* **70**, 572-581, (2014).

5 Schuck, P. Size-distribution analysis of macromolecules by sedimentation velocity ultracentrifugation and lamm equation modeling. *Biophys. J.* **78**, 1606-1619, (2000).

6 Ji, X. *et al.* Three-dimensional structure, catalytic properties, and evolution of a sigma class glutathione transferase from squid, a progenitor of the lens S-crystallins of cephalopods. *Biochemistry* **34**, 5317-5328, (1995).

**Extended Data Table 1**

**Summary of crystallographic information for octopus S-crystallin Q108F mutant**

| Data Collection | |
|---|---|
| Space group | $P6_422$ |
| Cell dimensions | |
| *a*, *b*, *c* (Å) | 114.3, 114.3, 63.9 |
| α, β, γ (°) | 90, 90, 120 |
| Resolution[a] (Å) | 30-2.35 (2.43-2.35) |
| $R_{merge}$[b] (%) | 16.4 (53.2) |
| $I / \sigma I$ | 13.3 (4.0) |
| Completeness (%) | 99.9 (100.0) |
| Redundancy | 7.1 (7.2) |

| Refinement | |
|---|---|
| Number of reflections | 10,177 (1,251) |
| *R* factor[c] (%) | 19.5 |
| Free *R* factor[d] (%) | 25.0 |
| Number of atoms | 1,782 |
| Protein | 1,713 |
| Ligand/ion | 20/5 |
| Water | 44 |
| *B*-factors | |
| Protein | 24.7 |
| Ligand/ion | 23.5/28.7 |
| Water | 26.6 |
| rmsd | |
| Bond length (Å) | 0.009 |
| Bond angles (°) | 1.3 |
| Ramachandran analysis[e] (%) | |
| Favored | 91.4 |
| Allowed | 8.6 |
| Disallowed | 0 |

[a] The numbers in parentheses are for the highest-resolution shell.

[b] $R_{merge} = \sum_h \sum_i \left| I_{hi} - \langle I_h \rangle \right| / \sum_h \sum_i I_{hi}$ , where $I_{hi}$ is the integrated intensity of a given reflection and $\langle I_h \rangle$ is the mean intensity of multiple corresponding symmetry-related reflections.

[c] $R = \sum_h \left| F_h^o - F_h^c \right| / \sum_h F_h^o$ , where $F_h^o$ and $F_h^c$ are the observed and calculated

structure factors, respectively.

[d] Free *R* is *R* calculated using a random 5% of data excluded from the refinement.

**Extended Data Table 2**

**Primer used for making the S-crystallin mutants**

| Primer | Sequence (5' to 3') |
|---|---|
| R43K-F | CAGAATGGGACAGCATGAAAAACAAGATGCCATGTCA |
| R43F-R | TGACATGGCATCTTGTTTTTCATGCTGTCCCATTCTG |
| H49N-F | ATGAGAAACAAGATGCCATGTAACATGATGCCAATGTTGG |
| H49N-R | CCAACATTGGCATCATGTTACATGGCATCTTGTTTCTCAT |
| Q64A-F | AACAGAACCCAAATTCCCGCGAGTATGGCTATGGCCAG |
| Q64A-R | TGGCCATAGCCATACTCGCGGGAATTTGGGTTCTGTTG |
| L100F-F | CAGACTGCTTCTATGACATCTTTGACGATTACATGAGAA |
| L100F-R | TTCTCATGTAATCGTCAAAGATGTCATAGAAGCAGTCTG |
| D101A-F | TGCTTCTATGACATCTTGGCTGATTACATGAGAATGTAC |
| D101A-R | GTACATTCTCATGTAATCAGCCAAGATGTCATAGAAGCA |
| D101N-F | GCTTCTATGACATCTTGAACGATTACATGAGAATG |
| D101N-R | CATTCTCATGTAATCGTTCAAGATGTCATAGAAGC |
| M104V-F | GACATGTTGGACGATTACGTGAGAATGTACCAGGATG |
| M104V-R | CATCCTGGTACATTCTCACGTAATCGTCCAAGATGTC |
| Q108F-F | GACGATTACATGAGAATGTACTTCGATGGTAACTGCAGAATG ATG |
| Q108F-R | CATCATTCTGCAGTTACCATCGAAGTACATTCTCATGTAATCG TC |
| C112G-F | ATGTACCAGGATGGTAACGGCAGAATGATGTTCCAGCGA |
| C112G-R | TCGCTGGAACATCATTCTGCCGTTACCATCCTGGTACAT |
| L100F/D101N-F | GACTGCTTCTATGACATCTTCAACGATTACATGAGAATGTAC |
| L100F/D101N-R | GTACATTCTCATGTAATCGTTGAAGATGTCATAGAAGCAGTC |
| M104V/Q108F-F | GACATCTTGGACGATTACGTGAGAATGTACTTCGATGGT |
| M104V/Q108F-R | ACCATCGAAGTACATTCTCACGTAATCGTCCAAGATGTC |
| L100F/D101N/M 104V-F | GACTGCTTCTATGACATCTTCAACGATTACGTGAGAATGTAC |
| L100F/D101N/M 104V-R | GTACATTCTCACGTAATCGTTGAAGATGTCATAGAAGCAGTC |
| Δloop(112-122)-F | ATGTACCAGGATGGTAACAGCAGCTCCTCTGAGAAG |
| Δloop(112-122)-R | CTTCTCAGAGGAGCTGCTGTTACCATCCTGGTACAT |
| Δloop(112-122)/Q 108F-F | GATTACATGAGAATGTACTTCGATGGTAACAGCAGCTCC |
| Δloop(112-122)/Q 108F-R | GGAGCTGCTGTTACCATCGAAGTACATTCTCATGTAATC |

**Extended Data Table 3**

**Steady-state kinetic parameters of S-crystallin mutants**

| Protein[a] | [Protein] (μM) | $K_{m,GSH}$ (mM)[c] | $K_{m,CDNB}$ (mM)[c] | $k_{cat}$ ($s^{-1}$)[c] |
|---|---|---|---|---|
| R43K | 0.3 | 0.23 ± 0.07 | 1.3 ± 0.1 | 0.40 ± 0.01 |
| H49N | 0.5 | 0.13 ± 0.02 | 5.1 ± 1.8 | 0.60 ± 0.14 |
| Q64A | 5.4 | 6.3 ± 0.5 | unsaturated | 0.03 ± 0.003 |
| L100F | 0.07 | 2.5 ± 0.3 | 2.8 ± 0.4 | 3.7 ± 0.3 |
| D101A | 0.4 | 2.6 ± 0.5 | 2.2 ± 0.6 | 1.2 ± 0.2 |
| D101N | 0.5 | 0.4 ± 0.04 | 2.5 ± 0.6 | 0.41 ± 0.05 |
| M104V | 0.09 | 1.1 ± 0.1 | 0.65 ± 0.07 | 2.3 ± 0.1 |
| Q108F | 0.02 | 8.2 ± 0.8 | unsaturated | 10.6 ± 0.6 |
| C112G | 1.7 | 0.02 ± 0.002 | unsaturated | 0.08 ± 0.002 |
| L100F/Q108F | 0.02 | 3.7 ± 0.5 | 1.4 ± 0.2 | 10.8 ± 0.5 |
| D101A/Q108F | 0.03 | 4.1 ± 0.5 | 5.3 ± 1.2 | 24.3 ± 3.5 |
| D101N/Q108F | 0.01 | 5.1 ± 0.5 | 0.67 ± 0.11 | 13.4 ± 0.6 |
| M104V/Q108F | 0.02 | 10.0 ± 1.6 | 4.1 ± 0.7 | 11.6 ± 1.1 |
| L100F/M104V/Q108F | 0.02 | 3.7 ± 0.4 | 2.4 ± 0.3 | 8.5 ± 0.5 |
| Δloop[b] | 0.6 | 2.2 ± 0.2 | 3.3 ± 0.6 | 0.38 ± 0.04 |
| Δloop/L100F | 0.07 | 5.7 ± 0.9 | 4.5 ± 1.0 | 2.0 ± 0.3 |
| Δloop/D101N | 1.1 | 5.3 ± 0.6 | 3.9 ± 0.7 | 0.4 ± 0.04 |
| Δloop/M104V | 0.7 | 6.1 ± 1.3 | 2.3 ± 0.5 | 0.32 ± 0.02 |
| Δloop/Q108F | 0.05 | 6.2 ± 0.9 | 1.9 ± 0.5 | 5.2 ± 0.4 |

[a] For GSH titration, the concentration of CDNB was 3.5 mM. For CDNB titration, the concentration of GSH was 10 mM.

[b] The loop between α4 and α5 helices (residue 112-122) was deleted and is designated as Δloop.

[c] Data were fitted to the Michaelis-Menten equation and the $R_{sqr}$ values were 0.980 to 0.999, respectively. All the assays were repeated at least twice to ensure reproducibility.

**Extended Data Table 4**

**Thermal stability of S-crystallin mutants with or without 1 mM GSH**

| Protein[a] | $T_m$ (°C) | $T_m$ in GSH (°C) | $\Delta T_m1$ (mutant-WT) | $\Delta T_m2$ (in GSH-apoform) |
|---|---|---|---|---|
| L100F | 42.8 ± 1.7 | 48.1 ± 2.6 | -5.4 | 5.3 |
| D101N | 45.5 ± 1.1 | 48.8 ± 1.4 | -2.7 | 3.3 |
| M104V | 45.1 ± 1.2 | 47.4 ± 1.9 | -3.1 | 2.3 |
| Q108F | 49.1 ± 0.7 | 48.8 ± 1.4 | 0.9 | -0.3 |
| C112G | 50.7 ± 0.7 | 58.2 ± 2.2 | 2.5 | 7.5 |
| L100F/Q108F | 43.7 ± 0.9 | 43.6 ± 1.3 | -4.5 | -0.1 |
| D101N/Q108F | 45.2 ± 1.3 | 46.3 ± 1.4 | -3.0 | 1.1 |
| M104V/Q108F | 44.1 ± 1.7 | 44.8 ± 1.7 | -4.1 | 0.7 |
| L100F/M104V/Q108F | 41.1 ± 0.6 | 43.3 ± 1.8 | -7.1 | 2.1 |
| Δloop | 46.9 ± 0.9 | 46.5 ± 1.4 | -1.3 | -0.4 |
| Δloop/L100F | 45.5 ± 1.3 | 45.2 ± 1.5 | -2.7 | -0.3 |
| Δloop/D101N | 43.1 ± 1.2 | 45.4 ± 1.5 | -5.1 | 2.3 |
| Δloop/M104V | 45.9 ± 0.8 | 46.7 ± 1.9 | -2.3 | 0.8 |
| Δloop/Q108F | 43.5 ± 0.9 | 43.1 ± 1.8 | -4.7 | -0.4 |

[a] The protein concentration was at 7.2 μM. The ellipticity at 222 nm was monitored at varying temperature ranging from 25 to 85°C. The results were fitted to the two-state unfolding model to calculate $T_m$ of S-crystallin.

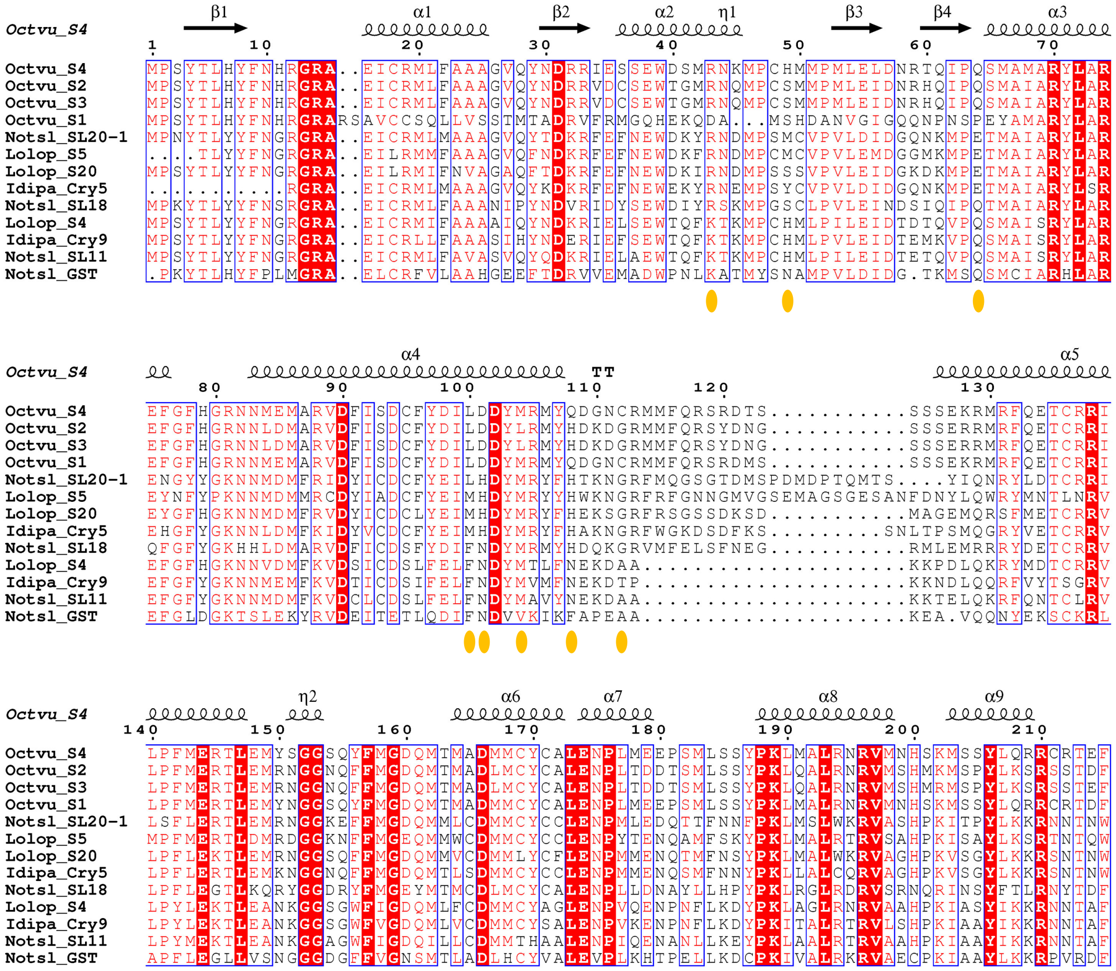


**Extended Data Fig. 1.** Sequence alignment of S-crystallins and GST-σ from cephalopods. Modified from an output from ESPript[1]. For comparison, Octvu_S4 shares 52-82% amino acid sequence identity with Octvu_S2 (accession number: P27014), S3 (Q25626), S1 (P27013), Notsl_SL20-1 (P18425), Lolop_S5 (Q25359), Lolop_S20 (Q25371), Idipa_Cry5 (A0A0H5ANU6), Lolop_S4 (Q25357), Notsl_SL18 (P27016; residues 1-120 and 214-308 were used), Idipa_Cry9 (A0A0H5ARE1), Notsl_SL11 (P18426) and 38% amino acid sequence identity with Notsl_GST (1GSQ_A), respectively. Orange ovals indicate some important residues in the active site that have been mutated in the present studies.

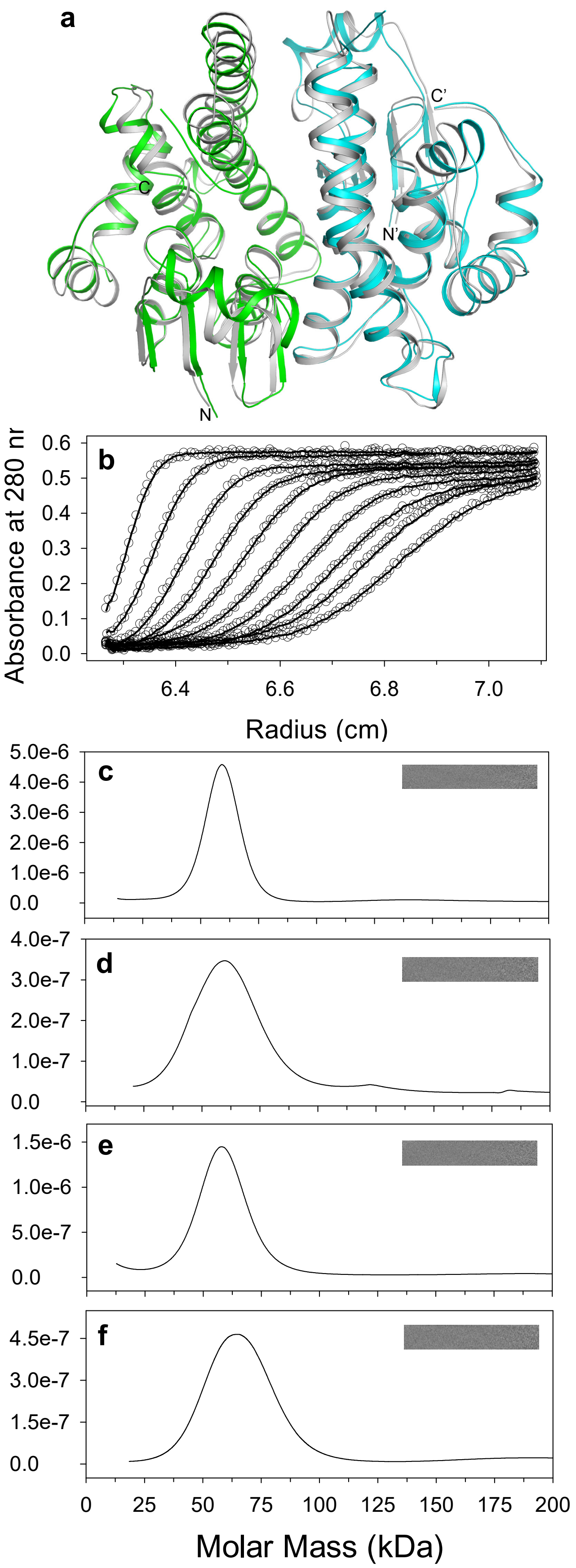


**Extended Data Fig. 2.** The octopus S-crystallin forms a stable dimer. (a) Overlay of

the dimeric S-crystallin and GST-σ. The dimers show similar orientation. (b) Traces of absorbance at 280 nm of the S-crystallin in 100 mM phosphate buffer (pH 6.5) during the sedimentation-velocity experiment[2-4] by analytical ultracentrifugation. The protein concentration was 1 mg/ml. For clarity, only every two scan is shown. The circles represent experimental data and the lines are the results after fitting to the Lamm equation using SEDFIT[5]. (c-f) The continuous c(M) distribution of S-crystallin and its mutants, L100F/D101N, ΔLoop/L100F/D101N and L100F/D101N/M104V/Q108F, all show a major species located at ~60 kDa. It indicates that it is a dimer as the molar mass of monomeric S-crystallin is 28 kDa. The residual bitmaps of the raw data and the best-fit result are shown as the gray bar. The protein concentration for the distribution analysis was 0.025 mg/ml (0.9 μM).

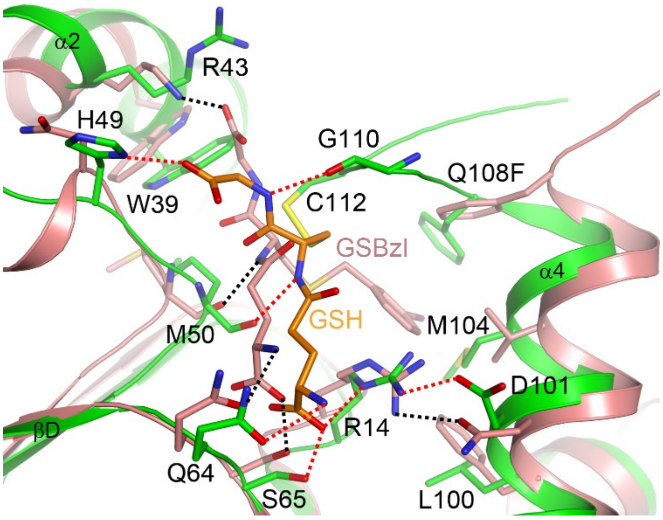


**Extended Data Fig. 3.** Overlay of the active sites of octopus S-crystallin Q108F mutant in complex with GSH (colored by green) and GST-σ in complex with S-(3-iodobenzyl) glutathione (GSBzI) (colored by salmon)[6]. The iodobenzyl ring of GSBzI is located in the hydrophobic pocket consisted by the residues Phe98, Val102 and Phe106 of GST-σ, while the equivalent residues in S-crystallin are Leu100, Met104 and Gln108, respectively.

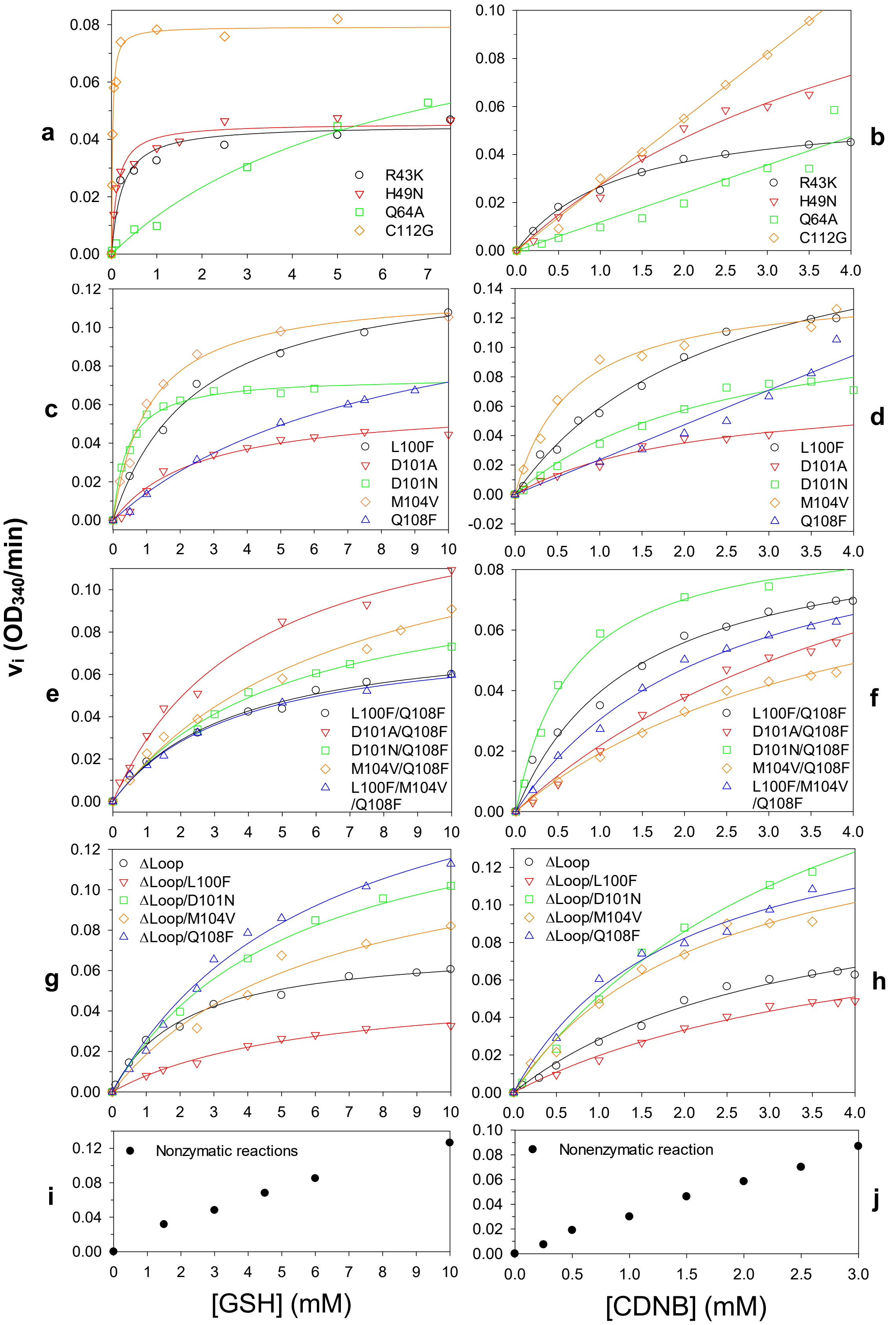


**Extended Data Fig. 4.** The GST activity of various S-crystallin mutants. Plots of the initial velocity as a function of the concentration of the two substrates GSH (a, c, e, g) and CDNB (b, d, f, h) for S-crystallin mutants and nonenzymatic reactions (i, j) were

shown, respectively. The solid lines are the best-fit by the Michaelis-Menten equation. The kinetic parameters such as $K_m$ and $k_{cat}$ from the best fit were shown in Table 1 and Extended data Table 3.

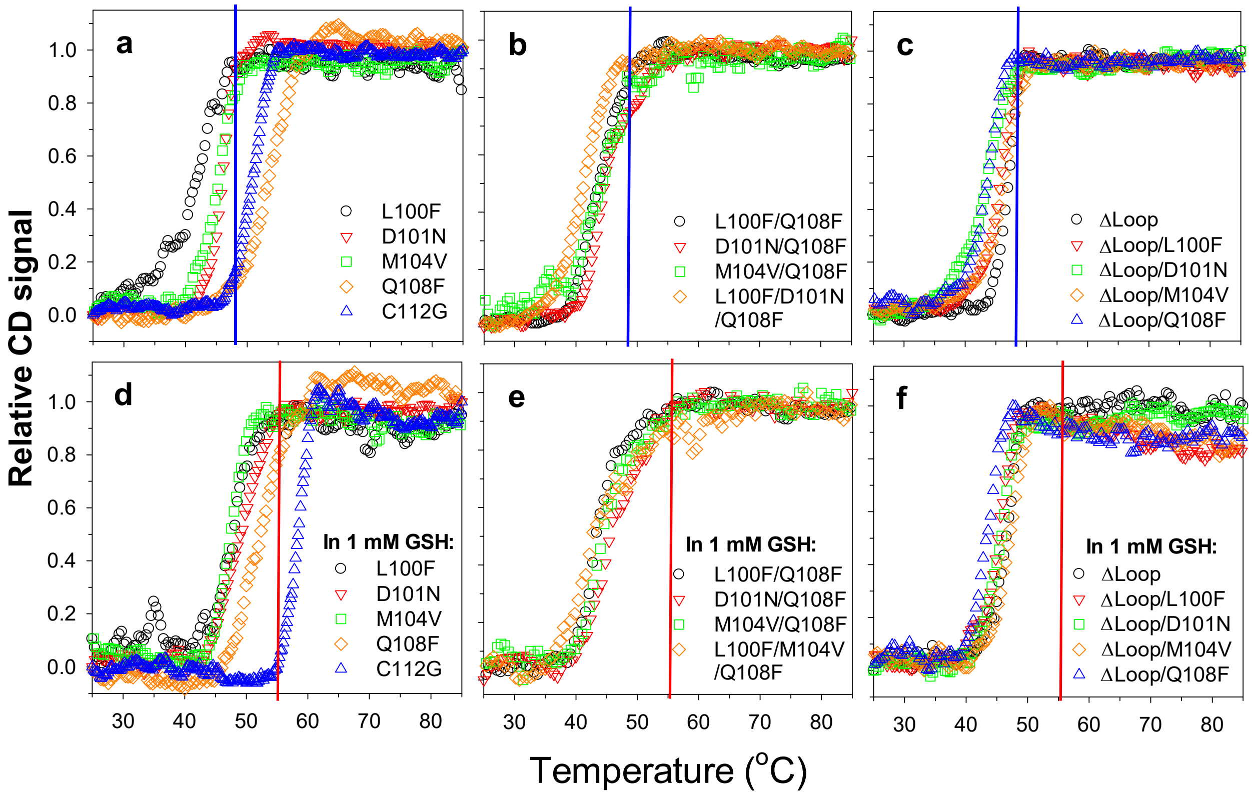


**Extended Data Fig. 5.** Thermal stability of S-crystallin mutants without (a-c) and with (d-f) 1 mM GSH by circular dichroism spectroscopy. Plot of the relative CD signal from the ellipticity at 222 nm as a function of the temperature for the S-crystallin mutants, respectively. The protein concentration was at 7.2 μM. The results were fitted to calculate the $T_m$, which are shown in Table 2. For comparison, the blue and red lines showed the $T_m$ of wild-type S-crystallin without and with 1 mM GSH, respectively.

a

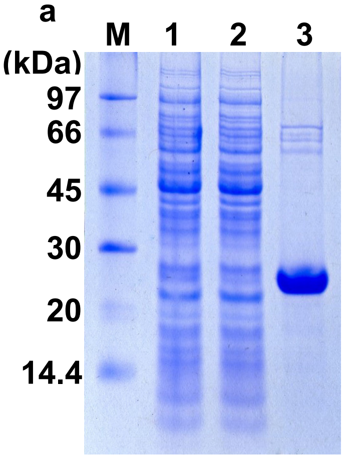


b

Individual ions scores > 5 indicate identity or extensive homology (p<0.05).

| Start - End | Observed | Mass | Sequence |
|---|---|---|---|
| 4 - 14 | 699.35 | 1396.69 | YTLHYFPLMGR (ion score 55) |
| 15 - 32 | 688.67 | 2063.00 | AELCRFVLAAHGEEFTDR (ion score 3) |
| 20 - 32 | 497.91 | 1490.71 | FVLAAHGEEFTDR (ion score 105) |
| 20 - 43 | 925.46 | 2773.35 | FVLAAHGEEFTDRVVEMADWPNLK (ion score 64) |
| 33 - 43 | 434.55 | 1300.64 | VVEMADWPNLK (ion score 75) |
| 33 - 60 | 1037.17 | 3108.50 | VVEMADWPNLKATMYSNAMPVLDIDGTK (ion score 54) |
| 44 - 60 | 609.62 | 1825.85 | ATMYSNAMPVLDIDGTK(ion score 109) |
| 74 - 80 | 383.19 | 764.36 | EFGLDGK (ion score 31) |
| 74 - 85 | 662.34 | 1322.67 | EFGLDGKTSLEK (ion score 60) |
| 81 - 87 | 299.49 | 895.47 | TSLEKYR(ion score 22) |
| 86 - 104 | 1148.57 | 2295.12 | YRVDEITETLQDILDDVMK (ion score 117) |
| 88 - 104 | 659.66 | 1975.96 | VDEITETLQDILDDVMK (ion score 117) |
| 113 - 117 | 356.66 | 711.31 | MMFQR (ion score 36) |
| 120 - 132 | 498.57 | 1492.71 | DAAKEAVQQNYEK (ion score 27) |
| 124 - 132 | 554.76 | 1107.51 | EAVQQNYEK (ion score 21) |
| 177 - 183 | 419.24 | 836.47 | HTPELLK (ion score 41) |
| 177 - 187 | 427.56 | 1279.66 | HTPELLKDCPK (Ions score 11) |
| 201 - 207 | 269.50 | 805.50 | IAAYLKK (ion score 30) |
| 208 - 213 | 263.81 | 788.43 | RPVRDF (ion score 17) |

c

1 MPK**YTLHYFP** **LMGRAELCRF** **VLAAHGEEFT** **DRVVEMADWP** **NLKATMYSNA**
51 **MPVLDIDGTK** MSQSMCIARH LAR**EFGLDGK** **TSLEKYRVDE** **ITETLQDILD**
101 **DVMK**IKQAPE CR**MMFQR**SR**D** **AAKEAVQQNY** **EK**SCKRLAPF LEGLLVSNGG
151 GDGFFVGNSM TLADLHCYVA LEVPLK**HTPE** **LLKDCPK**IVA LRKRVAECPK
201 **IAAYLKKRPV** **RDF**

**Extended Data Fig. 6.** Expression and purification of recombinant "S-crystallin"-like GST. (a) Protein identification by SDS-PAGE. M: molecular marker. Lane 1-3: cytoplasmic fraction, flow through, elution from the nickel affinity column. (b-c) Protein sequence identification by mass spectrometry. The protein was digested by trypsin and then analyzed by LC-MS/MS spectrometry. There are 19 matched peptides observed (b) and 61% sequence coverage are shown in bold red (c). Underlines show the point mutations and loop insertion.
